# Feasibility of a randomized controlled trial of functional strength training for people between six months and five years after stroke: FeSTivaLS trial

**DOI:** 10.1186/1745-6215-15-322

**Published:** 2014-08-12

**Authors:** Kathryn Mares, Jane Cross, Allan Clark, Susan Vaughan, Garry R Barton, Fiona Poland, Kate McGlashan, Martin Watson, Phyo K Myint, Marie-Luce O’Driscoll, Valerie M Pomeroy

**Affiliations:** School of Rehabilitation Sciences, University of East Anglia, Norwich Research Park, Queen’s Building, Norwich, NR4 7TJ UK; Norwich Medical School and Norwich Clinical Trials Unit, University of East Anglia, Norwich, NR4 7TJ UK; Norwich Medical School, University of East Anglia, Norwich, NR4 7TJ UK; Colman Centre for Specialist Rehabilitation Services, Unthank Road, Norwich, NR2 2PJ UK; School of Medicine & Dentistry, University of Aberdeen, Polwarth Building, Foresterhill, Aberdeen, AB25 2ZD UK; Department of Sports Therapy and Physiotherapy, Faculty of Health and Social Sciences, University of Bedfordshire, Park Square, Luton, LU1 3JU UK; UK and Norwich Medical School, University of East Anglia, Norwich, NR4 7TJ UK

**Keywords:** Stroke, Rehabilitation, Walking, Upper extremity, Physical therapy, Exercise, Functional strength training

## Abstract

**Background:**

Functional Strength Training (FST) could enhance recovery late after stroke. The aim of this study was to evaluate the feasibility of a subsequent fully powered, randomized controlled trial.

**Methods:**

The study was designed as a randomized, observer-blind trial. Both interventions were provided for up to one hour a day, four days a week, for six weeks. Evaluation points were before randomization (baseline), after six weeks intervention (outcome), and six weeks thereafter (follow-up). The study took place in participants’ own homes. Participants (n = 52) were a mean of 24.4 months after stroke with a mean age of 68.3 years with 67.3% male. All had difficulty using their paretic upper (UL) and lower limb (LL). Participants were allocated to FST-UL or FST-LL by an independent randomization service. The outcome measures were recruitment rate, attrition rate, practicality of recruitment strategies, occurrence of adverse reactions, acceptability of FST, and estimation of sample size for a subsequent trial. Primary clinical efficacy outcomes were the Action Research Arm Test (ARAT) and the Functional Ambulation Categories (FAC). Analysis was conducted using descriptive statistics and thematic analysis of participants’ views of FST. A power calculation used estimates of clinical efficacy variance to estimate sample size for a subsequent trial.

**Results:**

The screening process identified 1,127 stroke survivors of whom 52 (4.6%) were recruited. The recruitment rate was higher for referral from community therapists than for systematic identification of people discharged from an acute stroke unit. The attrition rate was 15.5% at the outcome and follow-up time-points. None of the participants experienced an adverse reaction. The participants who remained in the study at outcome had received 68% of the total possible amount of therapy. Participants reported that their experience of FST provided a sense of purpose and involvement and increased their confidence in performing activities. The power calculation provides estimation that 150 participants in each group will be required for a subsequent clinical trial.

**Conclusions:**

This study found that a subsequent clinical trial was feasible with modifications to the recruitment strategy to be used.

**Trial registration:**

Controlled-trials.com ISCTN71632550, 30 January 2009.

## Background

People often experience permanent disability after stroke that impacts adversely on everyday life. Indeed, stroke leaves about 66% of survivors with long-term limb impairments [1]. This is an unsatisfactory outcome that could be ameliorated by the provision of task-specific re-training of everyday function [2]. Participation in functional re-training, however, requires the production of sufficient voluntary activation of paretic muscle to attain the muscle strength thresholds required for everyday activity [3]. Strength training is therefore also used after stroke and has been found to enhance recovery [4, 5] and maintain this for up to four years after stroke [6]. There are potentially even better effects if strength training is combined with task-specific training [3]. Initial evidence suggests that functional strength training (FST) compared with rehabilitation as usual could enhance upper limb (UL) recovery [7] for people up to three months after stroke, although it might provide little advantage for the lower limb (LL) [8], except for habitual gait speed [9]. FST has also been found to enhance walking function when compared with no intervention in people who are at least one year after stroke and able to walk 10 m independently [10]. It is unknown whether or not FST might enhance the motor function of people who are unable to walk 10 m independently and are in the so-called chronic stage after stroke.

The emergent hypothesis is that people who are six months or more after stroke improve motor function in response to FST. The first step in testing this hypothesis is presented in this article, which reports a feasibility study as a precursor to a subsequent clinical trial [11–13]. The specific objectives were: to estimate the recruitment and attrition rates for a subsequent clinical trial; to assess and refine the practicality of a participant recruitment strategy for use in a subsequent clinical trial; to assess the occurrence of potential adverse reactions to FST provided for people in their homes between six months and five years after stroke; to assess the acceptability of delivering FST to stroke survivors in their homes through (a) eliciting participants’ opinions of their expectations and experiences and (b) recording fidelity to the intervention protocol; to inform a power calculation to estimate the sample size for a subsequent clinical trial with data of clinical efficacy, and its variance, of FST to enhance upper and lower limb motor function in people who are six months to five years after stroke; and to explore the feasibility of collecting resource use and quality of life data to inform the design of the health economics component of a future definitive trial.

## Methods

A summary of the methods is given here. A full description is given in the published protocol [14].

### Design, setting, randomization, and ethics

The design was a two-group, randomized, observer-blind, feasibility study based in participants’ own homes with an embedded qualitative investigation of participants’ expectations and experiences of FST. The assessor, who conducted the efficacy and health economics measurement battery at baseline, outcome, and follow-up time-points, remained blinded to participants’ group allocation throughout the trial. A research therapist provided participants with their allocated intervention for up to one hour a day, four days a week, for six weeks. Measures were repeated on completion of the intervention phase (outcome) and six weeks thereafter (follow-up).

Participants were recruited from the discharge database of one acute stroke service, the six-month post-stroke clinic of the same stroke service, and therapist referral. After providing informed consent, participants undertook the measurement battery (baseline). An independent randomization service concealed group allocation until contacted by a researcher, and then used the baseline scores for the Functional Ambulation Category (FAC) [15] and Action Research Arm Test (ARAT) [16] to minimize any imbalance in allocation of participants to either FST-UL or FST-LL. In this study the FAC was categorized as: mild (score of 4 or more - walks independently on level ground but requires assistance with for example, stairs and slopes), moderate (score of 3 - requires the verbal supervision and/or stand-by help of one person), or severe (score of 2 or less - continuous or intermittent assistance of one person required). The ARAT was categorized as: mild (score between 39 and 57, when 57 indicates normal completion of all items), moderate (score between 20 and 38, when 38 equates to ability to complete all items albeit slowly and/or abnormally), or severe (score between 0 and 19, when 19 indicates the ability to complete all items in part). Allocating participants to one of two experimental groups, FST-UL or FST-LL, was used to minimize the potential confounder of one group receiving less therapy than the other and an inactive therapy [14]. Although some clinicians highlighted to the researchers that they expected a cross-training effect between the upper and lower limbs, such an interaction is not supported by clinical research evidence [14, 17].

Ethical approval was granted by the Norfolk Ethics Committee (reference number 09 H0308 147). The Current Controlled Trials registration identifier is ISRCTN71632550.

### Study population and sample size

Study criteria were similar to those in our earlier studies conducted with people who were early after stroke [7, 8]: aged 18 years and over, between six months and five years after a stroke (infarct or hemorrhage) in the anterior circulation (anterior or middle cerebral artery); able to walk four steps with support from one person and/or an assistive device, but in 15 seconds unable to step on and off a 7.5 cm high block, with either leg, more than 14 times (Step Test) [18]; able to move the paretic hand from lap to table surface, but unable to pick up £1 coins individually and stack four in an even pile; able to follow a one-stage command with the non-paretic upper limb; no known pathology contraindicating participation in FST; and not participating in formal upper or lower limb physical therapy.

To provide data for a subsequent sample size calculation we required at least 30 participants to estimate parameters of interest [19] as hypothesis testing is not the focus of a feasibility study [13]. In order to provide some assurance that our sample size would provide sufficient data for a power calculation, a preliminary power calculation estimated that 26 participants per group would have 90% power at 5% significance (two-tailed) to detect a change of: 1 point on the FAC [15] with the assumption that the standard deviation (SD) would be 1, and 5.7 points on the ARAT [16], with the assumption that the SD would be 5.7 [20].

For exploration of the expectations and experiences of undertaking FST we used purposive sampling [21] to recruit six of the participants to an embedded qualitative investigation. The purposive selection criteria were people receiving FST-UL and those receiving FST-LL, both women and men, participants across the age range represented in the study cohort, and participants with different ability in functional use of the paretic upper and lower limb.

### Functional strength training

FST provided in the present study has been previously described [7, 8, 14]. In essence, it involves repetitive progressive resistive exercise during functional task-specific training [7, 8, 14]. Examples of FST-UL exercises used in this trial include variations of: reaching, picking up a jug containing water and pouring contents into a container; picking up a container and removing the screw lid; reaching down to a foot and then using both hands to lace up a shoe; and picking up and then moving everyday objects of various weights and sizes to position them in a different locations of diverse heights.

Examples of FST-LL exercises used in this trial include variations of: standing up and sitting down; ascending and descending stairs and/or using a block for step up/step down exercise; practice of balance activity including one-leg standing; and walking whilst avoiding and/or stepping over obstacles.

Activities were progressed systematically, increasing the amount of resistance and number of repetitions. Resistance was varied using external resistance bands and/or weights and also increasing task difficulty through strategies such as decreasing seat height for sit or stand activities and increasing or decreasing the requirement for hand grip span. Progression was informed by the Oxford program [22]. This provided a framework for advancing the strengthening program. In practice, as the objects used to create loading were often functional items such as bottles, or functional tasks such as sit-to-stand, the therapist judged when the participant was easily able to achieve ten repetitions and would then increase the load or the difficulty of the task so that the strengthening program was progressed. If participants became fatigued, which was assessed as an increasing difficulty in performing the activity and/or self-reported feelings of tiredness, the therapist initially changed activities or offered a rest period until either one-hour of therapy was completed or it became apparent that the participant was unable to continue with the intervention that day.

### Participants’ expectations and experiences of undertaking functional strength training

An experienced qualitative researcher undertook semi-structured audio-taped interviews with participants in their own homes, at the baseline and outcome time-points. The full interview schedule is available in the published protocol [14]. In summary, they used indicative questions to explore participants’ stories of their life before the stroke, the experience of having a stroke, thoughts about the recovery process, their expectations of FST (baseline only), and experiences of participating in FST (outcome only). Participants were encouraged to raise issues they perceived to be pertinent about their stroke, their recovery, their lives, and the FST they received.

### Outcomes

Data were collected to calculate the recruitment and attrition rates, assess the productivity of recruitment strategies, monitor adverse reactions in both groups, identify and record participants’ expectations and experiences, and assess the total amount (hours) of FST received by participants compared to the total possible amount.

For adverse reactions it was specifically postulated that paretic limb pain could occur if FST was provided in a dose (amount in hours) that was too much for a participant. Pain was considered an adverse reaction if the therapist providing FST received a verbal or behavioral report of pain on four consecutive treatment days. Paretic limb pain was included in the monitoring as it was highlighted to the research team that this was a specific clinical concern.

For the cost-effectiveness aim of this feasibility study, we employed a purpose-designed cost questionnaire (these have been submitted to the Database of Instruments for Resource Use Measurement) and the EuroQol-5D (EQ-5D) [23]. These assessments were undertaken by participants at the baseline, outcome, and follow-up time-points.

Sample size for a subsequent clinical trial was estimated using a power calculation informed by clinical efficacy data. The primary outcomes were the FAC [15] for lower limb function and the ARAT [16] for upper limb function. Secondary outcomes were the Modified Rivermead Mobility Index (MRMI) [24] and the Timed Up and Go Test (TUG) [25] to assess mobility, and the Nine Hole Peg Test (9HPT) [26] to assess hand dexterity.

### Analysis

The recruitment rate was calculated as the percentage of people screened who were eligible for the study and subsequently provided informed consent. The attrition rate was calculated as the percentage of those recruited who did not undertake the measurement battery at the outcome and follow-up time-points. The practicality of the recruitment strategies was judged by consideration of the recruitment rate of each strategy.

The occurrence of potential adverse reactions to FST was judged by the percentage of participants who experienced one or more adverse reactions. This was analyzed for the whole sample and by allocation group. The acceptability of FST to participants was assessed by considering the participant’s’ opinions of their expectations and experiences of FST together with the percentage of the possible total amount of FST that was received by participants.

Information for estimation of the sample size for a subsequent clinical trial was provided by calculation of the clinical efficacy, and its variance, of FST-LL and FST-UL. First, a full-case analysis was performed in which all individuals with data at the outcome time-point were included according to the assigned treatment group. Second, an intention-to-treat analysis with all individuals included was conducted by imputing the values for those missing. The method for imputing the missing data was iteratively chained equations with all outcome measures included, prognostic baseline factors, and treatment group [27].

ARAT data was analyzed by ranking each individual and comparing the mean rank, based on a regression model with the minimization variables included, between the two groups. The *P*-value and confidence interval were estimated using the non-parametric bootstrap [28]. FAC categories were compared using the proportional odds model with group and minimization variables included. The proportional odds model assumption was tested. FAC categories were further categorized to ensure that sufficient numbers were included in each category; the classification was 0 to 1, 2 to 3, and 4 to 5. The MRMI data were compared between groups using the same approach outlined above for ARAT. The TUG data were analyzed by firstly comparing those individuals who could complete the task, and then by comparing the time using a log-transformed linear regression model including group and the minimization variables. A logarithmic transformation was used as the TUG data was positively skewed. Data for the 9HPT were analyzed by comparing the number of people in each group who could complete all 9 pegs in 50 seconds on at least one of three attempts.

To estimate the parameters needed for a formal sample size calculation for a subsequent pragmatic trial, the variation in outcome measure was estimated from the primary analysis and the recruitment and attrition rates predicted from those in this trial. The analysis was undertaken according to the predefined statistical analysis plan, agreed with the Trial Steering Committee prior to the unblinding of the data. Hence the differences in the specific statistical tests as described in the published protocol [14]. The feasibility of collecting resource use and quality of life data was assessed via the completion rates for the cost-questionnaires and EQ-5D at each of the time points.

### Study management

A Trial Steering Committee (TSC) provided oversight of this feasibility study. The TSC Chair was independent of the research team. Meetings of the TSC were held throughout the course of the study to ensure adherence to Good Clinical Practice requirements and to monitor recruitment, attrition, and adverse reactions to FST. As this was a feasibility study, a formal data monitoring committee was not convened.

### Service user involvement

A local stroke service users forum reviewed a protocol for this feasibility study and their comments were incorporated into the final version submitted for external peer-reviewed research funding. The stroke service users welcomed investigation of therapy provided in peoples’ own homes and had no concerns about the intervention. Once research funding was obtained, ongoing public involvement was provided by the Patient and Public Involvement in Research Group (PPIRES, South Norfolk Clinical Commissioning Group, UK.).

## Results

### Baseline characteristics of participants

Table 1 provides participants’ characteristics. In summary, participants were a mean of 24.4 months after stroke with a mean age of 68.3 years. The median score for ARAT was 15.7 (total possible = 57) and for FAC was 2.5 (total possible = 5). All characteristics were balanced across the two groups except for stroke classification. The FST-UL group had a higher percentage of people clinically classified as having a partial anterior circulation stroke and the FST-LL had a higher percentage of people as having a lacunar stoke or posterior circulatory stroke (see discussion section relating to recruitment strategy feasibility).

**Table 1:** **Baseline characteristics of all randomized participants**

	FST-UL (n = 27)	FST-LL (n = 25)
Age in years*	67.6 (12.9)	69.0 (13.7)
Months since stroke*	24.4 (16.6)	24.4 (13.7)
Gender^#^		
Male	18 (66.7)	17 (68.0)
Female	9 (33.3)	8 (32.0)
Hemiside^#^		
Left	15 (55.6)	12 (48.0)
Right	12 (44.4)	13 (52.0)
Stroke classification^#^		
LACS	6 (22.2)	9 (36.0)
PACS	12 (44.5)	9 (36.0)
TACS	3 (11.1)	6 (24.0)
POCS	3 (11.1)	1 (4.0)
hemorrhage	3 (11.1)	0 (0)
Action Research Arm Test^+^	16.7 (13.5)	14.8 (12.9)
Able to complete Nine Hole Peg Test^#^	1 (3.9)	0 (0)
Functional Ambulation Categories^#^		
0	3 (11.1)	2 (8.0)
1	4 (14.8)	5 (20.0)
2	8 (29.6)	7 (28.0)
3	1 (3.7)	2 (8.0)
4	11 (40.7)	9 (36.0)
5	0	0
Modified Rivermead Mobility Index^+^	26.8 (3.4)	26.1 (4.4)
Timed Up and Go Test*	42.3 (31.5)	49.3 (36.8)

*mean (standard deviation); ^#^number of participants (%); ^+^median (interquartile range). FST-UL = Functional Strength Training for Upper Limb; FST-LL = Functional Strength Training for Lower Limb; LACS = lacunar anterior circulation stroke; PACS = partial anterior circulation stroke; ST-UL = Functional Strength Training for Upper Limb; TACS = total anterior circulation stroke.

The six participants involved in the qualitative investigation were in the age range of 41 to 80 years, three were male, and four were allocated to FST-LL. Full details are provided in Table 2.

**Table 2:** **Characteristics of participants in the qualitative investigation of their expectations and experiences of FST**

Gender	Age group (years)	FST group	Household type	Residential area type
Male	51-60	FST-UL	Single, living with friends	Urban estate
Male	51-60	FST-LL	Married, living with wife	Rural
Female	41-50	FST-LL	Separated, living with partner	Rural town
Female	71-80	FST-LL	Single, living alone	Rural town
Female	71-80	FST-LL	Widowed, living alone	Rural town
Male	51-60	FST-UL	Divorced, living with lodgers	Suburban

### Recruitment and attrition

**Figure 1:**
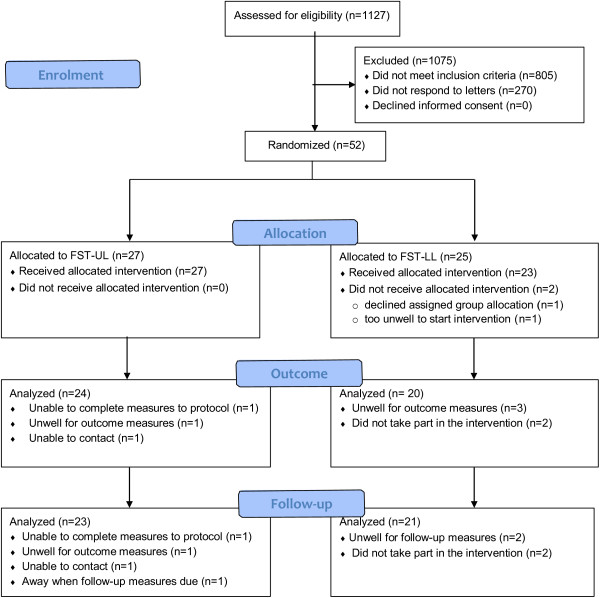
**Trial flowchart.** FST-UL = functional strength training for the upper limb; FST-LL = functional strength training for the lower limb.

Recruitment took place over a two-year period (March 2010 to March 2012). The first participant was recruited on 4 March 2010, with the final participant being recruited on 19 March 2012 and the final follow-up measure taken on 19 July 2012.The CONSORT flowchart gives details of screening, recruitment, and attrition during this feasibility trial (Figure 1). In brief, 1,127 stroke survivors were assessed for eligibility and 1,075 were excluded. The remaining 52 provided informed consent and were randomized, providing a recruitment rate of 4.6%.

Twenty-seven participants were allocated to FST-UL with all receiving their allocated intervention, and 25 to FST-LL with 23 receiving their allocated intervention (one person declined the allocated intervention and one became unwell). Attrition rates were 11% and 15% for FST-UL and 20% and 16% for FST-LL at outcome and at follow-up, respectively. The mean attrition rate at outcome was therefore 15.5% at outcome and follow-up for the entire sample.

### Practicality of participant recruitment strategies

After the first four months of recruitment it became apparent that the study was not meeting the intended recruitment rate. Two people had been recruited whilst the target was eight by that point. Consequently, the decision was made to also recruit participants via referrals from both the stroke Early Supported Discharge team (ESD) and from therapists providing healthcare for stroke survivors in other parts of community services (therapist referral). The majority of participants were recruited via therapist referral (69%) whilst no referrals were made from the ESD. Other measures to improve the recruitment rate included securing funding from the Comprehensive Local Research Network for increased administration support in order to increase the number of invitation letters that were sent out. This secured 27% of referrals, with the remaining 4% evenly distributed between referral via a friend who had already participated in the study and a poster that had been displayed in a local clinical setting.

Although the recruitment rate was increased with the therapist referral strategy for those participants it was not possible to access the neuroimaging information in a timely way to confirm stroke location. Therefore, inclusion or otherwise was based on clinical presentation. This resulted in the inclusion of four participants, subsequently identified to have a stroke affecting the posterior circulation (Table 1).

### Occurrence of adverse reactions to FST

None of the participants experienced an adverse reaction.

### Acceptability of FST

All participants received the intervention as allocated except one participant who withdrew from the FST-LL group as he wanted FST-UL. The content of FST-UL and FST-LL was consistent with the protocol (Table 3) and the amount of therapy was essentially the same in the two groups (Table 4).

**Table 3:** **Content of functional strength training for participants who completed both baseline and outcome measures: percentage of total therapy time**

	Group allocation	
	FST-UL	FST-LL
FST-UL provided	54.5	NA
Functional movement training – upper limb	26.0	NA
Focus primarily on resistance during function	8.7	NA
Facilitation upper limb activity from another body part	5.6	NA
Focus on cueing	5.2	NA
Gravity-neutral repetitive movement		
FST-LL provided	NA	67.8
Functional movement training – lower limb	NA	13.8
Focus primarily on resistance during function	NA	10.9
Performance of specific movement patterns	NA	7.5
Promotion muscle activity and joint movement during function	54.5	NA

FST-UL = Functional Strength Training for Upper Limb; FST-LL = Functional Strength Training for Lower Limb; NA = not applicable.

**Table 4:** **Functional strength training for participants who completed both baseline and outcome measures: time-duration and reasons for missing entire sessions**

	Allocated group	
	FST-UL	FST-LL
Hours of FST-UL for 24 participants		
Total delivered	410.2	NA
Total planned	576.0	NA
Percentage of planned that was delivered	71.3%	NA
Mean delivered per participant	17.1	NA
Hours of FST-LL for 19 participants		
Total delivered	NA	325.7
Total planned	NA	504.0
Percentage of planned that was delivered	NA	64.6%
Mean delivered per participant	NA	17.1
Reasons for missed sessions (% of planned sessions)		
Participant unwell	2.8%	2.4%
Participant cancelled	5.4%	7.5%
Therapist unavailable	2.8%	3.6%
Annual leave/bank holiday	0.7%	1.1%
Data unavailable	0%	0.8%

FST-UL = Functional Strength Training for Upper Limb; FST-LL = Functional Strength Training for Lower Limb; NA = not applicable.

The intervention was embraced by qualitative study participants without exception. They expressed entirely positive views about the potential benefits it might bring to their lives. They viewed participation in the study as a way of accessing further rehabilitation which they perceived as lacking since their discharge from hospital. Two major themes emerged from the data regarding the experience of FST; these concerned an increase in their confidence regarding activities and a sense of purpose and involvement, as one man commented about goal setting:

‘I need someone saying P you have got to do this and take my arm and put it where it ought to be and say this is what you are trying to get to’ (male participant P152).

Most of these perceptions seemed to be conditional on the characteristics of those therapists who delivered the intervention, as with their listening skills:

‘I have tried to do this and I just can’t do this and they listen to you and then you know you would just keep going’ (female participant P126).

But there was some evidence of their developing increased self-efficacy that persisted into the follow-up data, for instance, in being able to plan and then to realize these plans:

‘I’ve been thinking about getting into town but now I’ve got more confidence to do it’ (female participant P141).

### Sample size for subsequent clinical trial

Table 5 displays the results for the primary and secondary outcomes. There was a significant difference in the ARAT scores between the groups, with the lower limb group having a lower score at both outcome (*P* = 0.042) and at follow-up (*P* = 0.019). The effect sizes were -5.06 (95% CI -9.93 to –0.18) and -5.91 (95% CI -10.85,to –0.97) respectively. There was no difference in the FAC scores between the groups at either outcome (*P* = 0.654) or at follow-up (*P* = 0.925). The proportional odds assumption for FAC was tested and no reason was identified to reject the fit of the model (outcome: *P* = 0.964; follow-up: *P* = 0.821). The only other outcome to show a significant difference was the TUG at outcome (*P* = 0.047), with the upper limb group completing the test in a shorter time (effect size 1.61 (95% CI 1.01 to 2.59). In addition, fewer individuals in the lower-limb group could complete the TUG (100% and 80% respectively) although this was not significantly different (*P* = 0.169). The results of the imputed data, in terms of the effect sizes and significance levels, are similar to those of the observed and are therefore not presented.

**Table 5:** **Outcome scores over time for both groups**

		Outcome						Follow-up					
		FST-UL (n = 24)		FST-LL (n = 25)		Effect size for difference between groups	*P*-value ^+^	FST-UL (n = 23)		FST-LL (n = 21)		Effect size	*P*-value
		N	Mean (SD)/N (%)	N	Mean (SD)/N (%)			N	Mean (SD)/N (%)	N	Mean (SD)/N (%)		
**Primary outcomes**													
ARAT		24	22.9 (14.2)	19	14.2 (14.0)	-5.06 (-9.93,-0.18)^*^	0.042	23	21.0 (14.9)	20	15.6 (14.2)	-5.91 (-10.85,-0.97)^*^	0.019
FAC		24		20				23		21			
	0		3 (12.5)		1 (5.0)	0.73 (0.18,2.89)^#^	0.654		4 (17.4)		2 (9.5)	0.94 (0.24,3.69)^#^	0.925
	1		2 (8.3)		6 (30.0)				3 (13.0)		5 (23.8)		
	2		5 (20.8)		4 (20.0)				4 (17.4)		4 (19.1)		
	3		1 (4.2)		0 (0.0)				0 (0.0)		2 (9.5)		
	4		13 (54.2)		9 (45.0)				12 (52.2)		8 (38.1)		
**Secondary outcomes**													
MRMI^*^		19	26.5 (4.7)	19	26.4 (2.0)	-3.2 (-10.0,3.7)^*^	0.367	18	27.3 (3.1)	20	26.5 (3.9)	0.49 (-4.75,5.72)^*^	0.856
TUG - time (secs)^1^		20	26.1 (17.1)	19	47.1 (35.0)	1.61 (1.01,2.59)^+^	0.047	19	37.1 (30.3)	18	39.8 (28.3)	0.93 (0.72,1.21)^§^	0.592
TUG - ability to complete ^2^		24	20 (82.6)	20	19 (90.5)	5.35 (0.49,58.21)	0.169	23	19 (82.6)	20	17 (85.0)	1.53 (0.25,9.35)	0.648
9HPT - ability to complete^2^		24	1 (4.2)	17	0 (0.00)	NA	1.000^3^	23	1 (4.4)	0	0(0.00)	NA	1.000^3^

^+^ *P*-value for difference between treatment groups; *mean difference in rank; ^#^common odds ratio for a one unit increase; ^§^ratio of means;; ^1^the average of those times taken to complete the task; ^2^the ability to complete the task at least once; ^3^based on Fisher’s exact test ignoring factors used in minimization. 9HPT = Nine Hole Peg Test; ARAT = Action Research Arm Test; FAC = Functional Ambulation Categories; FST-LL = Functional Strength Training - Lower Limb; FST-UL = Functional Strength Training - Upper Limb; MRMI = Modified Rivermead Mobility Index; TUG = Time Up and Go Test;

Using the standard deviation data from ARAT and FAC scores from this trial and the dropout rate, the estimated sample size to provide 90% power at 5% significance for a subsequent multicenter trial is 150 participants per group to detect a 5.7 unit change in ARAT, and 57 per group to detect a 1.0 unit change in FAC.

### Completion rates for the cost-questionnaires and EQ-5D

At baseline, all of the LL and UL participants completed both the cost questionnaire and EQ-5D. At outcome, 21 out of 25 of the LL participants (84.0%) completed both of these measures, compared to 24 out of 27 UL participants (88.9%). The same rates were achieved at follow-up. Additionally, the therapists who delivered the FST intervention provided data for consultation times for all of the LL and UL participants.

## Discussion

This feasibility study has provided important information for a subsequent clinical trial. Design of a subsequent trial will be informed by the estimates from current findings of a recruitment rate of 5%, and attrition rates of 11% for FST-UL and 20% for FST-LL at the primary time-point after the six-week intervention phase. The findings reported here indicate that the recruitment strategy for a subsequent trial needs to consider that referral from therapists might produce the greatest yield, however a process for accessing neuroimaging data in a timely manner is needed in order to confirm the location of the stroke. None of the participants experienced an adverse reaction to FST and whilst this does not provide robust evidence of safety, when considered with the data from participant interviews, it does suggest that delivery of FST to people late after stroke in their own homes is practical. Incorporating health economics measures in a subsequent trial is likely to provide sufficient data as both the cost questionnaire and EQ5D were completed by 84% of the FST-LL and 89% of the FST-UL participants. The sample size for a subsequent trial is estimated, based on actual data from this feasibility trial, as 150 participants per group.

The recruitment rate of 5% to the present trial was lower than the 9% and 10% of the two earlier trials of FST [7, 8]. The present trial recruited people who were living at home a mean of two years after stroke compared with people in an in-patient rehabilitation facility a mean of 20 [7] and 34 [8] days after stroke. Another potential influential factor could be that previous studies [7, 8] used face-to-face recruitment methods whereas we made first contact with people via a letter [14]. Recruitment rate to a subsequent trial might be higher, therefore, if face-to-face screening could be conducted. However, this is likely to be more costly in terms of travel to the homes of potential participants and researcher time. In addition, it would still require first contact to be via a letter in order to arrange a suitable visit time. Other strategies which may increase recruitment are: telephone reminders, use of opt-out procedures rather than opt-in, and participants knowing which intervention they receive [29]. Neither telephone reminders nor opt-out procedures were used in this feasibility trial. Opt-out procedures are difficult, if not impossible, to apply to exercise-based interventions and so this is unlikely to be a useful strategy. This leaves telephone reminders to non-respondents to the letters sent to inform stroke survivors about the trial, although this will require careful consideration in a research governance environment which safeguards people from unwanted intrusions. However, 63% of people surveyed about their attitudes to participation in clinical trials reported that they consider it appropriate to be contacted by telephone [30]. A potential way forward could be to conduct pretrial focus groups which have been found to provide useful information for the design of trial recruitment strategies [31].

A recruitment strategy used in a subsequent trial will need to consider the challenge encountered during this feasibility trial of ensuring timely access by research therapists to neuroimaging data held in the hospital hosting the acute stroke unit. This challenge arose when participants were recruited through referral from community-based therapists who did not have access to neuroimaging data or detailed medical notes. The process adopted was that used in clinical community practice whereby therapists make assessments based mainly on behavioral clinical presentation. This procedure resulted in the inclusion of four participants who were subsequently found to have stroke in the posterior not anterior circulation territory. This protocol deviation needs to be avoided. Any strategy needs to consider that a subsequent trial of FST in a community setting should recruit participants using criteria that can be replicated in clinical practice [32]. It is intended to explore this challenge with healthcare professionals working in stroke services in both acute and community settings.

A strong design aspect of the present trial is avoidance of the potential confounder of comparing experimental treatment to no treatment or to a conventional treatment of lower dose [33]. The potential confounding factor of treatment intensity [34] was also avoided.

Participants’ adherence to the planned interventions was greater than in the earlier trials. A mean of 17.1 hours of therapy for participants in the FST-UL group compared to 12.5 hours [7], and 17.1 hours for those in the FST-LL group compared to 14.8 hours [8]. This equates to 71% and 65% completion of intervention planned for FST-UL and FST-LL respectively. Interestingly, a study of a similar lower limb intervention with people who were a mean of 63 months after stroke reported 100% compliance with the planned intervention [10]. Although participants in the earlier study were all able to walk 10 m at baseline and the intervention dose was lower (30 minutes a day, three times a week for four weeks [10]), it remains possible that adherence to FST in this present feasibility trial could have been higher. In a subsequent trial, therefore, additional improvements in adherence will be sought through reinforcing emphasis on strategies to empower participants, such as shared decision-making for goal setting (including identification of motivation factors), providing personalized information to enhance an individual’s own responsibility for rehabilitation, and consideration of emotional states that could impact on participation [35, 36].

Acceptability of both forms of FST is evident from the data generated by interviews with participants in this feasibility trial. Indeed, only one participant withdrew from the trial for a reason directly related to treatment allocation and that was because of a preference for the other form of FST.

## Conclusions

With modifications to the protocol, particularly in respect of the recruitment strategy, this line of research can continue to a fully powered, randomized, controlled clinical trial. Pretrial focus groups with stroke survivors meeting the trial criteria will be held to inform a decision as to whether or not to use telephone reminders for non-respondents to invitation letters. In addition, consultation with health professionals working in stroke services in both acute and community settings will inform the procedure for ensuring that neuroimaging data is available in a timely manner to ensure precise characterization of people providing informed consent to confirm their enrolment into the trial. The other required modification to the protocol will strengthen the emphasis on strategies to empower individuals to participate in the FST interventions with a view to maximizing adherence to allocated therapy.

## Authors’ information

Susan Vaughan, Martin Watson and Marie-Luce O’Driscoll are no longer at the University of East Anglia. Only Marie-Luce O’Driscoll now has an academic institution affiliation and this is the University of Bedforshire, UK.

## Abbreviations

9HPT: Nine hole peg test
ARAT: Action Research Arm Test
FAC: Functional Ambulation Categories
FST: Functional strength training
IQR: Interquartile range
LACS: Lacunar anterior circulation stroke
LL: Lower limb
MRMI: Modified Rivermead Mobility Index
PACS: Partial anterior circulation stroke
POCS: Posterior circulation stroke
TACS: Total anterior circulation stroke
TUG: Timed Up and Go Test
UL: Upper limb.
